# Rotational Stability of Scaphoid Osteosyntheses: An In Vitro Comparison of Small Fragment Cannulated Screws to Novel Bone Screw Sets

**DOI:** 10.1371/journal.pone.0156080

**Published:** 2016-06-03

**Authors:** Jochen Erhart, Ewald Unger, Philip Schefzig, Peter Varga, Inga Trulson, Anna Gormasz, Alexander Trulson, Martin Reschl, Michael Hagmann, Vilmos Vecsei, Winfried Mayr

**Affiliations:** 1 Department of Trauma Surgery, Medical University of Vienna, Waehringer Guertel 18-20, A-1090, Vienna, Austria; 2 Center for Medical Physics and Biomedical Engineering, Medical University of Vienna, Waehringer Guertel 18-20, A-1090, Vienna, Austria; 3 AO Research Institute Davos, Clavadelerstrasse 8, CH-7270, Davos, Switzerland; 4 BG Clinic Tuebingen Schnarrenbergstr. 95, D-72076, Tuebingen, Germany; 5 Section for Medical Statistics, Center for Medical Statistics, Informatics, and Intelligent Systems, Medical University of Vienna, Spitalgasse 23, A-1090, Vienna, Austria; University of Zaragoza, SPAIN

## Abstract

**Background:**

The current standard of care for operative repair of scaphoid fractures involves reduction and internal fixation with a single headless compression screw. However, a compression screw in isolation does not necessarily control rotational stability at a fracture or nonunion site. The single screw provides rotational control through friction and bone interdigitation from compression at the fracture site. We hypothesize that osteosyntheses with novel bone screw sets (BSS) equipped with anti-rotational elements provide improved rotational stability.

**Methods:**

Stability of osteosynthesis under increasing cyclic torsional loading was investigated on osteotomized cadaveric scaphoids. Two novel prototype BSS, oblique type (BSS-obl.) and longitudinal type (BSS-long.) were compared to three conventional screws: Acutrak2^®^mini, HCS^®^3.0 and Twinfix^®^. Biomechanical tests were performed on scaphoids from single donors in paired comparison and analyzed by balanced incomplete random block design. Loading was increased by 50 mNm increments with 1,000 cycles per torque level and repeated until a rotational clearance of 10°. Primary outcome measure was the number of cycles to 10° clearance, secondary outcome measure was the maximum rotational clearance for each torque level.

**Findings:**

BSS-obl. performed significantly better than Acutrak2^®^mini and HCS^®^ (p = 0.015, p<0.0001). BSS-long. performed significantly better than HCS^®^ (p = 0.010). No significant difference in performance between BSS-obl. and BSS-long. (p = 0.361), between BSS obl. and Twinfix^®^ (p = 0.50) and BSS long. and Twinfix^®^ (p = 0.667) was detected. Within the torque range up to 200 mNm, four of 21 (19%) BSS-long. and four of 21 (19%) BSS-obl. preparations showed early failure. The same loading led to early failure in four (29%) Twinfix^®^, seven (50%) Acutrak2^®^mini and 10 (71%) HCS^®^ of 14 screw samples, respectively.

**Conclusions:**

For both BSS and to a lesser extent for Twinfix^®^ (as dual-component screw), higher rotational stabilities were identified in comparison to single component headless compression screws.

## Introduction

The objective regarding the therapy of scaphoid fractures is to obtain osseous consolidation as rapidly as possible in order to restore function of the wrist [1].

Even though surgical therapy by single screw osteosynthesis permits earlier return to work and exercise than conservative therapy, the difference in consolidation rate and speed of fracture healing itself is subject to controversial discussion [2,3,4]. Similarly to the different models on long bones, it is assumed that when delayed and non unions occur by shearing and torsion movements, newly formed tissues are torn, and the angioneogenesis in the fracture gap is interrupted, resulting in delayed or absent osseous healing of the bone [5,6,7]. These shearing and rotational movements between the scaphoid bone fragments originate in changing pressure conditions in the radiocarpal joint and between the carpal bones during motion and loading forces to the wrist [8,9].

It is not known if these shearing and torsion movements are possible causes of non union development and long consolidation times in conservative therapy. Compared to conservative treatment single screw osteosynthesis improved results in consolidation rate tremendously [3]. It is our suspicion that remaining non union development and extensive time for consolidation should also be ascribed to differing qualities of scaphoid bone osteosynthesis, e.g. the choice and positioning of implant. Mechanical tests are mandatory in order to evaluate specific biomechanical properties of different screw models and their handling. The experimental assessment of the stability and load bearing capacity of compression screw osteosyntheses utilizes primarily compression tests [10,11], pull-out and push-out tests in polyurethane foam (sawbone^®^) [12,13]; these, however, can only provide a vague idea of the efficacy of an in vivo osteosynthesis. Due to the special anatomical features of the scaphoid and its steering role within the structure and the mechanics of the carpus [14], rotation around the longitudinal axis of the fractured scaphoid is believed to be an important kinematic component influencing ossification [15,16,17]. The efficiency of osteosyntheses regarding the rotational stability of the scaphoid fragments about the longitudinal axis of the scaphoid is thus far insufficiently investigated [15,18]. When using conventional headless screws, the rotational stability of the scaphoid osteosynthesis is primarily determined by friction adhesion due to compression between the scaphoid bone fragments or by interlocking three-dimensionally formed fracture areas. However, some studies show that the compression force of the headless screws decreases over time, at least in vitro scaphoid bones [11,19], which must be accompanied by a reduction of the osteosynthesis stability, particularly against rotational strain in a twist-out direction of the screw. In clinical everyday routine, this problem is taken into account by blocking rotation using plate osteosyntheses [20] or through the additional use of Kirschner wires or a second scaphoid bone screw [8]. Greater efficiency of these methods is not proven thus far; due to its clinical proven advantage in terms of higher rate of osseous unions [3] and a shorter period for rehabilitation compared to conservative treatment [4] a singular compression screw osteosynthesis is justified indeed as the standard surgical procedure in stable and unstable fractures. To take rotational instability into account, we designed two bone screw sets (BSS) which do not substantially exceed the dimensions of the conventional compression screws at the fracture site and respectively consist of a first, compression screw and a second screw, which enters into a friction-lock connection with the first screw and blocks rotation of the fragments about its longitudinal axis. Apart from one study conducted on a sawbone model [18] no studies directly comparing rotational stability of osteosynthesized bone segments using different established headless screws are available in literature. We hypothesized that there are measureable differences in an in vitro test setup with scaphoids osteotomized at the waist in relation to different applied screw types. When selecting the screws, we intentionally emphasized second generation small fragment cannulated screws which had been in clinical use for longer periods and had different acting principles. In addition to tests with standard screws comparative tests with 2 types of custom designed bone screw set (BSS) prototypes with integrated blocking screw extensions were included.

## Methods

Acute evaluation of rotational stability of three different headless standard screw types in comparison to the two stabilizing prototype bone screw sets was performed in a balanced incomplete random block design experimental setup. The primary measure was the total number of stress cycles for each screw until a defined failure condition of a maximum rotational clearance bigger than 10° was reached. The secondary outcome was the mean rotational clearance for each screw.

**Fig 1:**
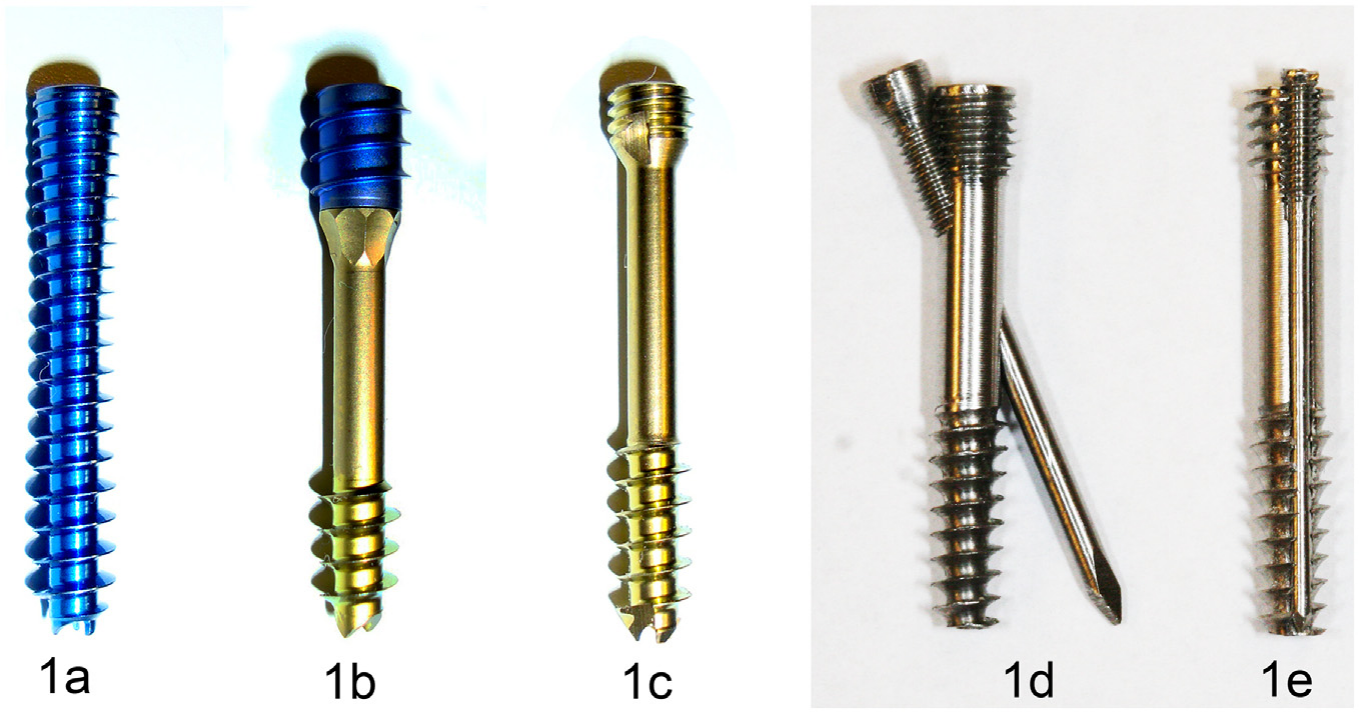
a-e. Screw types. The shown screws were used for the test series: Acutrak^®^2mini (Acumed) (Fig 1a), Twinfix^®^ (Stryker) (Fig 1b), HCS^®^ 3.0 (Synthes) (Fig 1c), and prototypes of the two new BSS sets with an additional crosswise drill hole with an inner thread in oblique type (BSS-obl.) (Fig 1d) and with a longitudinal groove with an inner thread and longitudinal screw as a longitudinal type (BSS-long.) (Fig 1e).

Fresh frozen cadaveric human scaphoid bones were obtained from donors who had signed a written informed consent form and voluntarily donated their bodies to the Center of Anatomy and Cell Biology for research. A total of 84 ex vivo scaphoid bones were used for the study. Three clinically established screw models:

Acutrak 2^®^ Mini Headless Compression Screw System, Acumed^®^, Hillsboro, OR, USA (Fig 1a), Twinfix^®^, Stryker^®^, Kalamazoo, Michigan, USA (Fig 1b), HCS^®^ 3.0 Countersinkable Compression Screw, (Synthes^®^, East West Chester, PA, USA) (Fig 1c), as well as 2 different prototypes of a specifically developed bone screw set (BSS), each consisting of 2 components, were used. (see Fig 1d and 1e). Both types of the bone screw set required targeting devices to place the second screw. Prototypes of both bone screw sets were made out of stainless steel 316L. All screws and screw sets measured a length of 22 mm. The screws were applied according to the manufacturer’s surgery instructions. Both variants of the BSS consisted of a primary compression screw as the first component and an additional secondary screw, which protrudes from the round cross-sectional profile of the primary compression screw in order to additionally block rotational movements of the scaphoid bone fragments about the primary screw axis.

### Scaphoid bone screw set oblique type (BSS obl.)

The primary screw of both BSS is a cannulated small fragment central threadless shaft screw with different pitches of the proximal and distal threaded portions of the screw. At the proximal end, there is a cylindrical thread with a maximum outer diameter of 3.5 mm and a gradient of 0.5 mm, over a length of 3 mm. The screw tip bears an 8.5 mm long conical thread with a gradient of 1 mm and an outer diameter which decreases from 3.2 to 2.6 mm towards the tip in a linear manner. This primary screw has an inner thread which is intended to receive the smaller secondary screw (24 mm long, countersunk head with a maximum outer diameter of 2.5 mm, cylindrical diameter of 1 mm, proximal thread with a diameter of 1.2 mm, 0.33 mm gradient and a length of 4.5 mm) with a crosswise centered drill hole which progresses at an angle of 20° to the longitudinal axis. In a composite with the secondary screw, this forms an intersecting rigid construction which stabilizes the connected bone segments against traction and torsion loads (Fig 1d).

### Scaphoid bone screw set longitudinal (BSS long.)

The second variant consists of an analogously designed compression screw, however—instead of the pass-through bore with a partly countersunk guiding channel (diameter 1 mm, 0.3 mm countersunk)—parallel to the longitudinal axis with a thread section near the base. The secondary torsion-stabilizing screw (22 mm long, headless, cylindrical 1 mm diameter, proximal thread with a diameter of 1.2 mm, 0.33 mm gradient, 3 mm in length) can be screwed into the countersunk guiding channel of the primary screw (Fig 1e).

## Experimental Set Up

Each of the two novel BSS was directly compared as individual reference screws to each of the standard screws. Left and right cadaver hands from one individual were used alternatively for a reference screw and a directly compared standard screw. The balanced incomplete random block design was chosen to account for differences in bone quality. To reduce the potential impact of training effect of the surgeon concerning the screw insertions, the different standard screws were applied in sequentially changing order from test-sequence to test-sequence. Seven test sequences of each standard screw type were performed in comparison with each of the two BSS types. Overall, 14 pieces of each of the three standard screws and 21 of each of the two BSS underwent the test procedure, summing up to the 84 scaphoid bones processed. Cyclical torque loads were applied under continuous monitoring of displacement between the two bone fragments.

The measuring procedure was defined in a manner that the osteotomized scaphoid bones which were being tested were stressed cyclically, with simultaneous measurement of the rotational clearance of the segments in relation to each other, each 1,000 times in twist-out direction at a torque level. Herein the base load equaled 50 mNm; stress was increased by 50 mNm for the further torque levels, with 1,000 cycles respectively. This process was continued until the termination criterion of 10° rotational clearance of the scaphoid bone fragments in relation to each other was reached. The testing time span, rotational clearance, torque and number of cycles were saved as a text file (ASCII^®^ data) and then statistically evaluated and graphically displayed using the table calculation program MS Excel (MS Office^®^ 2013, Redmont, USA). Aside from the primary and secondary outcome measure, this tool was also used to obtain the continuous progressions of the measurement figures of torque against rotational clearance.

### Technical set up

In order to design the test implementation for each ex vivo scaphoid bone in a standardized, reproducible manner, the following process protocol was specified for the orientation of clamping scaphoid bone fragments to the test array, the osteotomy and the osteosyntheses: High Resolution Peripheral Quantitative Computer Tomography (HR-pQCT, XtremeCT, Scanco^®^, Brutisellen Switzerland) scans were made of all ex vivo scaphoid bones which were used in the trial in order to verify the comparability of the bone density of the scaphoid bone pairs. Scan settings were 60 kVp energy, 1 mA current, 100 ms integration time, 1536x1536 pixels image matrix and 82 μm isotropic voxel size. The gray values of the voxels were converted to bone mineral density (BMD, HA mg/cm^3^) units using the calibration phantom data of the instrument. The images were coarsened by rescaling to 164 μm voxel size and the subchondral bone contour of each scaphoid was identified automatically by means of a 3D fill algorithm [21] using the software Medtool^®^ (www.dr-pahr.at/software). The mean and standard deviation of BMD was finally evaluated from the values of the voxels within this bone domain. Cadaveric scaphoids were excluded from analysis when apparent; intraosseous cysts were detected by HR-pQCT.

**Fig 2:**
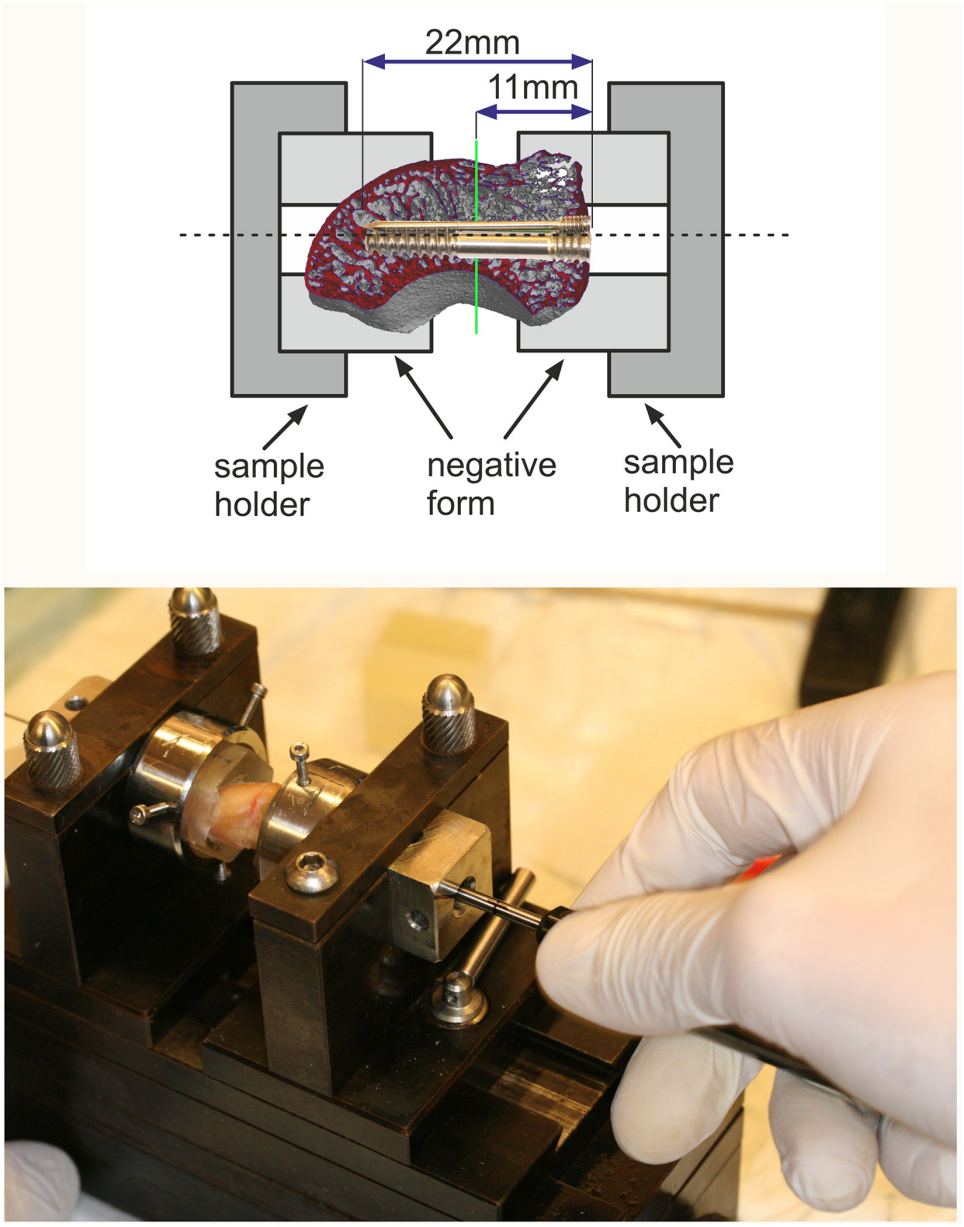
Preparation of the specimens. The scaphoid bones were clamped into the steel bracket in a fitted plastic form which was produced using the stereolithography technique. They were embedded in a device which permitted the correct orientation of the scaphoid bone for the osteotomy and screw insertion. In this illustration, the circular saw which was used for the osteotomy has already been removed, and the guide sheaths for the drill wire as well as the targeting drilling wire are visible. The osteosynthesized scaphoid bone was removed from the clamping device with its holders and clamped into the test array (Fig 3).

In particular the HR-pQCT data were used to digitally produce surface models via a surface reconstruction. Using the software Rhinoceros^®^ 4.0 (McNeel^®^ Europe, Barcelona, Spain) and 3D-Print (Objet Eden500V^®^, Rehovot, Israel), the surface models of each scaphoid bone were used to produce acrylic resin models as specific, precisely fitting negative forms of the scaphoid bones, which were glued to the defatted bone surface as a fixing base (Technovit^®^ 3040, Heraeus Kulzer GmbH, Wehrheim, Germany) and to connect them to the device for the osteotomy and osteosynthesis, as well as to the test array. This set up allowed to orient the test objects along the anatomical longitudinal axis of the scaphoid and clamp them in the sample holder in a defined manner to carry out the osteotomy and subsequent osteosynthesis (Fig 2). Using a circular saw, the osteotomy was placed orthogonally to the longitudinal axis, with the cutting plane in 11mm distance from the planned screw entry point, and within the central third of the scaphoid bone. Using ground openings of the negative forms and sample holders the samples were accessible to the instruments required for the osteosynthesis (Fig 2). A1 mm guide wire (Hofer-Medical^®^, Fuerstenfeld, Austria) to guide the drill and screw was positioned using a centering sheath. A specifically manufactured drill was used for the newly developed 2-component screw sets.

**Fig 3:**
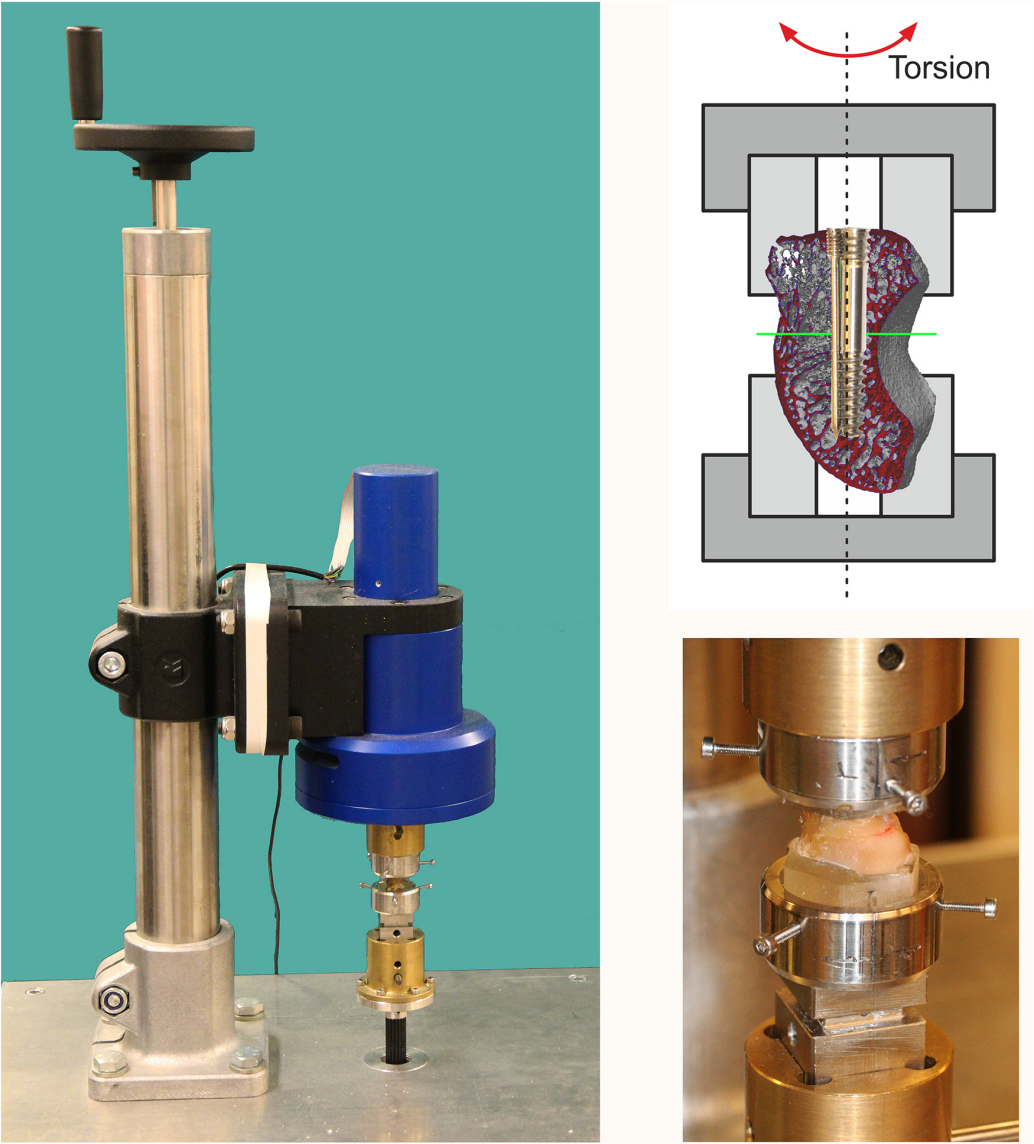
Test array. Draft of the test array for cyclic load to the scaphoid bone osteosyntheses, which were clamped into the holder.

The drill depth was limited to 24mm by markings on all drills. For the osteotomy, the fixing connections of the collet chuck were rigidly fixed against each other; after osteotomy the stops at the collet chuck were released for the subsequent osteosynthesis, so that the scaphoid bone fragments were stabilized against rotation, but unlocked and mobile in the axial direction to equalize distance. Repositioning was performed without diastasis between the scaphoid bone fragments. The osteosyntheses were implemented according to the manufacturer’s specifications, resulting in standardized compression conditions. The prototype screws were placed analogously to the instructions for the standard screws so that the screw end was precisely flush with the cortical surface. The samples and the sample holders were then taken out of the holder system and clamped into the test array for testing the rotational stability (see Fig 3).

The technical array was powered by a multiphase motor Type AM2224^®^ with a connected planetary drive 23/1 (Faulhaber^®^ GmbH, Schoeneich, Germany). The utilized torque was recorded and controlled through a feedback loop using an axially installed isometric torque sensor TS70^®^ and a measurement amplifier unit GSV3-LS^®^ (ME-Meßsysteme^®^ GmbH, Hennigsdorf, Germany). The drive and sensor were axially coupled to a clamping device for the fitted sample holder. On the side facing the screw tip, the torque sensor was rigidly connected to a base plate and bore the fitted part for the sample segment held by the screw tip on its upper end. The drive unit was mounted above, axially oriented and hanging in the array, and carried the fitted part for the sample end which was held by the proximal screw end. The sample holder on the screw end side was rigidly connected to the drive axle; the sample holder in the screw tip region was guided via a square bar (20mm) which permitted tension-avoiding equalization movements in the axial direction. This construction ensured that only rotational loads and not traction or pressure loads were introduced into the sample.

After the samples were mounted, the position of the unloaded sample holders with the osteosynthesized scaphoid bones which were fixed therein and the torque which was applied in the resting position were defined as the zero parameter via the test array control software. The multiphase motor was controlled via software controls and a serially connected motor driver (ADCM1S^®^, Faulhaber, Germany).

**Fig 4:**
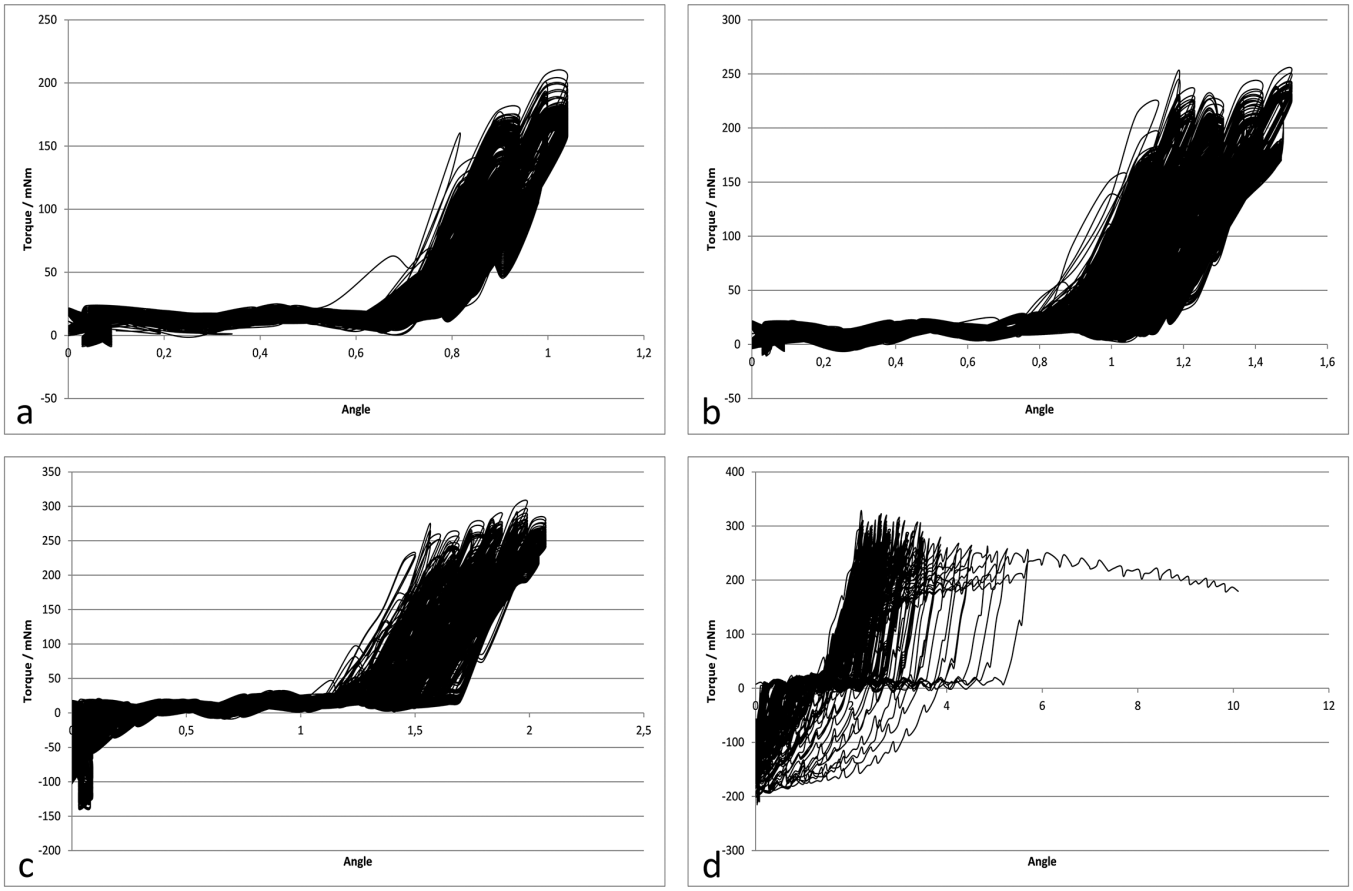
a-d. Sample of a force/angle diagram. Force/angle diagrams of the cyclic load of a scaphoid bone with a torque load from 150 to 300 mNm and resulting loss of stability. In Fig 4d loosening of the screw fastening is clearly visible by the curves which run out to the right and the line up to 10°.

The step size of the drive unit resulted from the gear ratio-controlled step width of the motor at 0.1°; the maximum attainable torque was 0.7 Nm. The measuring range of the TS70 torque sensor was limited to 10 Nm; the GSV3-LS amplifier unit provided digitalized measuring parameters with a sampling rate of 10 kHz and 10 bit (0.15 mNm) resolution. A specific control software was developed to operate the test array, carry out the automated control of the test processes and to record and visualize the measurement data. This software also continuously monitored rotational play and automatically stopped it when a maximum rotational clearance of 10° was reached (see Fig 4). The load was increased with 1,000 cycles, respectively, from 50 mNm up to 800 mNm in 50 mNm increments.

### Sample

Since no data on the variability of the proposed endpoint in our setting were available, the sample size considerations were based on similar experiments [15,16,22], where similar amounts of specimens were found sufficient to establish significant differences between different types of osteosynthesis methods or implants.

#### Statistical methods

Even though various studies [15,16] report symmetric bone density of contralateral scaphoids, we assumed possible density differences between contralateral scaphoid bones and therefore performed a Wilcoxon Signed Rank Test. The primary outcome (total numbers of stress cycles) was analyzed as with a mixed effects model that incorporates implant type (the different screws) as fixed-effect and body (both scaphoids of each donor are assumed to be more similar than scaphoids between donors) as a random effect. The 7 predefined comparisons (each of the established screws was compared with the two novel models and the novel models were also compared) were simultaneously tested as contrasts within formulated for this model. The results of these comparisons are presented along with least-squares estimates for the expectation of the number of cycles for each screw type. Adjustment for multiplicity was done by simulated Holm procedure. P-values less than 0.05 were regarded as significant.

The secondary outcome measure (rotational clearance) was modeled by a linear mixed effects model that incorporates implant type as fixed-effect, body as random-effect and stress level (torque) as a repeated factor. This model was used to estimate the expected maximum rotational clearance for each implant at each torque level. Therefore least squares estimate for the expected maximal rotational clearance for each implant and each torque level were calculated. Measurements were performed incrementally until the termination criterion of 10° rotational clearance of the scaphoid bone fragments in relation to each other; however, depiction took place up to 200 mNm, since we assumed greater clinical relevance in this lower torque range. No significance tests were conducted for this analysis. Descriptive statistics (mean, sd, quartiles and missings) for the raw outcomes are presented as numbers and in graphical form (box and dot plots).

This study was approved by the institutional ethics board of the Medical University of Vienna (Ethics Approval No.: 785/2008, Date: 01/13/2009, S1 Ethics Commission Statement).

## Results

We noted a statistically significant but practically irrelevant difference of the average density parameters between the right and left scaphoid bones (p = 0.04591). Right-hand scaphoid bones are denser on average (319.84±250.53 mg/cm^3^) than those on the left side (310.71±243.92 mg/cm^3^), wherein the observed minimum of the absolute parameters was at 119.46 mg/cm^3^ on the left and 74.94 mg/cm^3^ on the right, and its maximum was at 431.71 mg/cm^3^ on the left and 421.56 mg/cm^3^ on the right side (S1 Table Data Set A).

**Fig 5:**
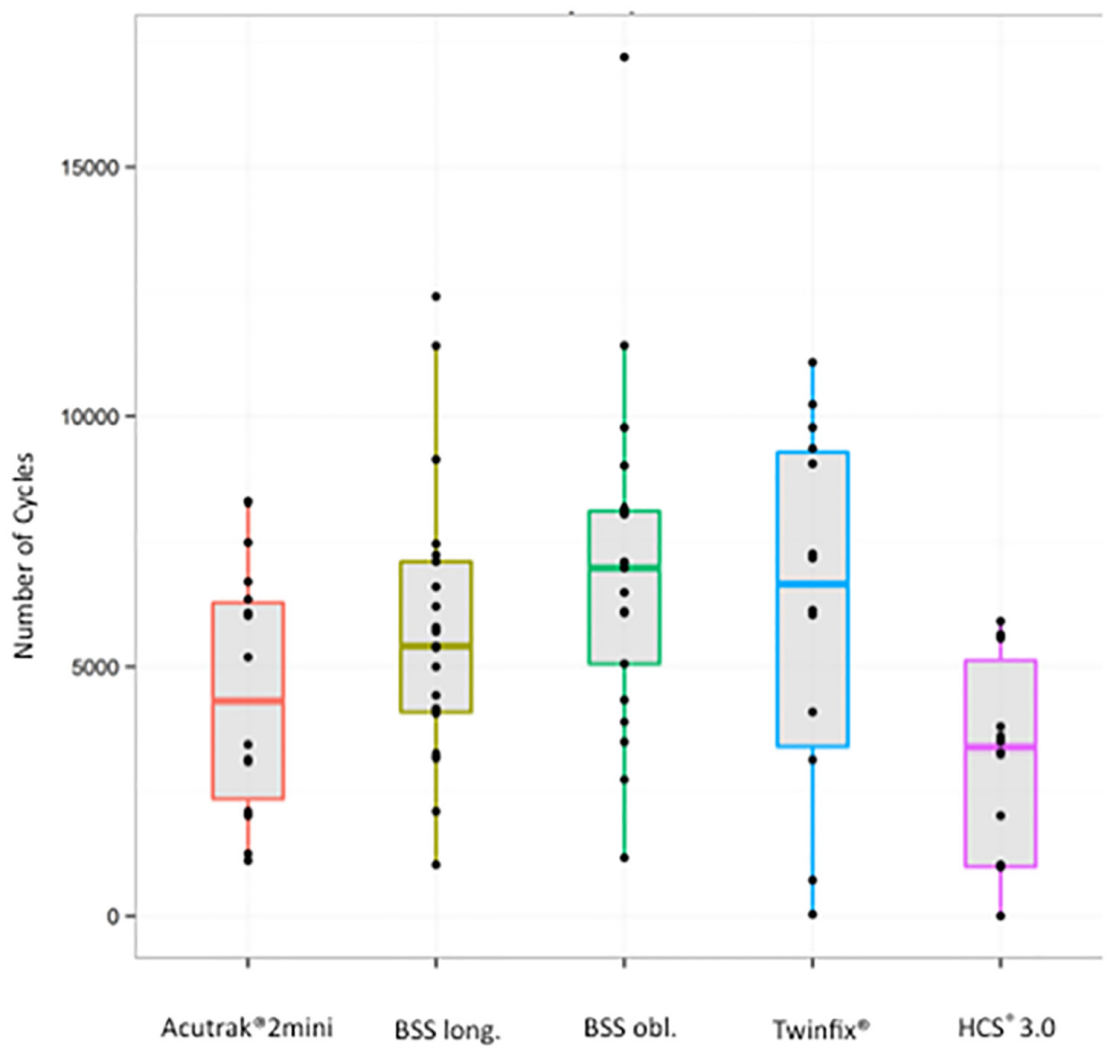
Primary outcome. Observed Cycles until failure by Implant. Median of maximum attained cycles of the respective screw models with average parameter and standard deviation. Box plot of maximum cycles per screw type. Median, 25% and 75% percentiles, the points correspond to the individual results per screw.

The results on the number of load cycles to the fault limit are shown in Table 1 and Fig 5, sorted by screw types.

**Table 1:** primary outcome (Statistics for primary outcome by implant): dataset on total cycles until failure for all tested screws.

Statistics for primary outcome by implant							
	Descriptives					LSM estimate	
Implant	min	perc25	med	perc75	max	mean	sd
**Acutrak 2 Mini**	1,080	2,086	4,302.5	6,332	8,280	4,347.37	741.03
**BSS, long. Type**	999	4,082	5,418	7,094	12,388	5,753.9	616.84
**BSS, obl. Type**	1,143	5,059	6,964	8,119	17,188	6,966.86	616.84
**Twinfix**	32	3,140	6,636.5	9,344	11,052	6,122.84	741.03
**HCS 3.0**	1	954	3,360.5	5,577	5,887	2,923.65	741.03

**Fig 6:**
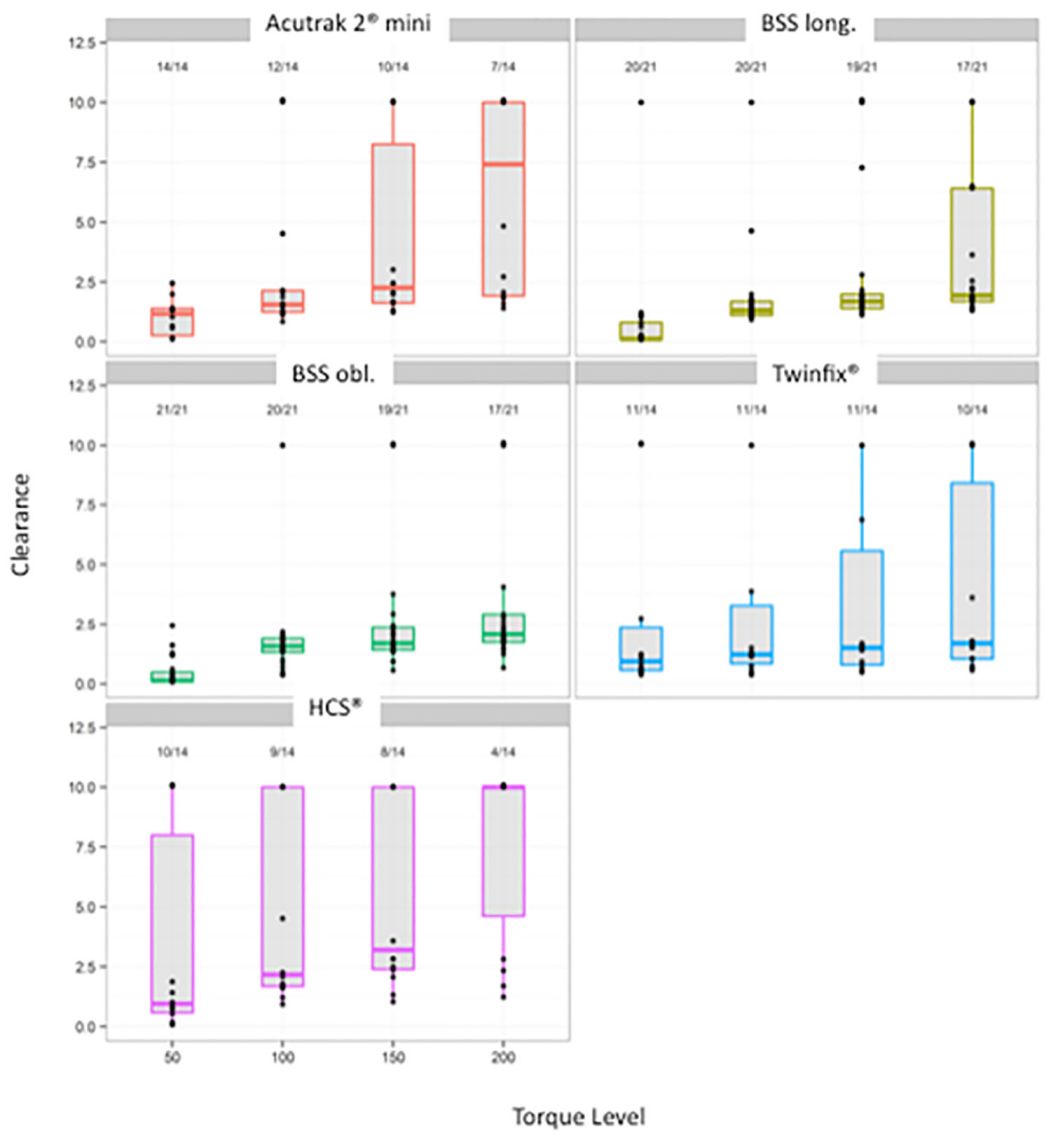
Secondary Outcome. Maximum rotational clearance at Torque level by Implant. Median of maximum attained cycles of the respective screw models with average parameter and standard deviation. Box plot of maximum displacement per screw type/load combination. Median, 25% and 75% percentiles, the points correspond to the individual results per screw. The number above the boxplots indicates the number of non-failing screws.

Further details by screw type and load stages are found in Table 2 and Fig 6, which are limited to the first four rotation stress cycles up to 200 mNm. (S2 Table Data Set B).

**Table 2:** Statistics for secondary outcome by implant. Adjustment for Multiplicity: Holm- Simulated.

Statistics for secondary outcome by implant								
Variable	Descriptives						LSM estimate	
	N	min	perc25	med	perc75	max	mean	Se
Implant = Acutrak 2 Mini (N = 14)								
Ang50	14	0.11	0.16	1.175	1.42	2.45	1.1324	0.7421
Ang100	12	0.85	1.23	1.555	2.16	10.1	2.9867	0.7421
Ang150	10	1.23	1.61	2.26	10	10.06	4.2867	0.7421
Ang200	7	1.4	1.88	7.415	10	10.1	6.241	0.7421
Implant = BSS, long. Type (N = 21)								
Ang50	20	0.09	0.1	0.12	0.81	10	0.4996	0.6283
Ang100	20	0.93	1.12	1.31	1.69	10	1.5901	0.6283
Ang150	19	1.12	1.4	1.69	1.99	10.1	2.3691	0.6283
Ang200	17	1.31	1.69	1.94	6.41	10.04	3.5305	0.6283
Implant = BSS, obl. Type (N = 21)								
Ang50	21	0.08	0.1	0.14	0.48	2.44	0.8085	0.6283
Ang100	20	0.37	1.32	1.58	1.89	10	2.2123	0.6283
Ang150	19	0.56	1.42	1.69	2.37	10.06	2.9438	0.6283
Ang200	17	0.67	1.74	2.07	2.92	10.1	3.9071	0.6283
Implant = Twinfix (N = 14)								
Ang50	11	0.37	0.56	0.94	2.74	10.08	2.951	0.7421
Ang100	11	0.37	0.75	1.225	3.88	10	3.1817	0.7421
Ang150	11	0.48	0.75	1.505	6.87	10	3.4839	0.7421
Ang200	10	0.56	1.04	1.69	10	10.08	3.9546	0.7421
Implant = HCS 3.0 (N = 14)								
Ang50	10	0.09	0.54	0.955	10.04	10.1	3.3002	0.7421
Ang100	9	0.93	1.69	2.17	10	10.04	4.7216	0.7421
Ang150	8	1.04	2.36	3.205	10	10.04	5.4887	0.7421
Ang200	4	1.23	2.82	10	10.03	10.1	7.6159	0.7421

For instance, an individual osteosynthesis with a BSS obl. reached 17,188 total cycles at 950 mNm (BMD of the sample 390.95 mg/cm^3^); another reached 11,397 at 650 mNm (BMD of the sample 431.71 mg/cm^3^). An individual BSS long. reached 12,388 at 700 mNm (BMD of the sample 380.51 mg/cm^3^). The best standard screw connection was reached by a single Twinfix^®^ screw with 11,052 at 650 mNm (BMD of the sample 381.23 mg/cm^3^).

The statistical analysis yielded the following results.

### Primary outcome—Cycles

The analysis shows a significant performance difference between implants regarding the primary outcome measure (p<0.0001, Table 3, Fig 5). The implant BSS obl. performed significantly better than the implants Acutrak^®^ 2 Mini and HCS^®^ 3.0 (p = 0.0149, p<0.0001). The implant BSS long. performed significantly better than HCS^®^ 3.0 (p = 0.0090). There was no significant difference in performance between BSS-obl. and BSS-long. (p = 0.361), between BSS obl. and Twinfix^®^ (p = 0.50) and BSS long and Twinfix^®^ (p = 0.667). No difference could be observed between BSS long. and Acutrak^®^ 2 Mini (p = 0.2959).

**Table 3:** primary outcome (Least squares means estimates for screw comparisons): dataset on cycles until failure with reference to torque loads (restricted to load range, where majority of failures occurred); N stands for number of screws which didn’t fail at a previous torque level and a further tested in the indicated load range.

Least Squares Means Estimates for Screw Comparisons Adjustment for Multiplicity: Holm-Simulated						
Comparison	Estimate	Standard Error	DF	t	p	adjusted p
BSS, long. type vs Acutrak 2 Mini	1,406.54	851.51	55.4	1.65	0.1042	0.2959
BSS, long. type vs Twinfix	-368.94	851.51	55.4	-0.43	0.6665	0.6665
BSS, long. type vs HCS 3.0	2,830.26	851.51	55.4	3.32	0.0016	0.009
BSS, obl. type vs Acutrak 2 Mini	2,619.49	851.51	55.4	3.08	0.0033	0.0149
BSS, obl. type vs Twinfix	844.01	851.51	55.4	0.99	0.3259	0.5038
BSS, obl. type vs HCS 3.0	4,043.21	851.51	55.4	4.75	<0.0001	<0.0001
BSS, obl. type vs BSS, long. type	1,212.96	821.3	63.48	1.48	0.1447	0.3065

### Secondary outcome—Cycles

The estimates and descriptive statistics are presented in Table 2. Fig 6 shows box plots for each screw and torque level. This figure shows the progression of rotational clearance with increased stress level for each implant, and the homogeneity of implant behavior. The two new BSS show a low progression in the rotational clearance and the implants for these types behave very similarly.

At torques of up to 200 mNm, the screw osteosyntheses showed differing average solidities (also see Fig 6). At 50 mNm torque, the termination criterion was reached in four out of 14 cases in the HCS^®^ 3.0 screw, in three out of 14 cases in the Twinfix^®^ screw and in one out of 21 cases in the BSS long. At 100 mNm torque, the termination criteria were reached in the HCS^®^ 3.0 screw in one further case, in the BSS obl. type for the first time in one case and in the Acutrak^®^ 2 Mini for the first time in two cases. No later than after 200 mNm torque, four of the 21 BSS long., four of the 21 BSS obl., four of the 14 Twinfix^®^, seven of the 14 Acutrak^®^ 2 Mini screws and 10 of the 14 HCS^®^ 3.0 screws reached the termination criterion.

## Discussion

Based on the targeted design of the BSS, we assumed differences in their mode of action and were indeed able to prove significant differences in rotational stability. The selected study design furthermore showed differences between the conventional screw types. Isolated osteosyntheses with different screw types were able to resist torque stress for a particularly large number of cycles into very high levels on the one hand; on the other hand osteosyntheses with the same conventional screws were noted as a result of some early failures. Early failures were observed in osteoporotic scaphoid bones as well as in bones of average bone density. The feasibility and usability of dual-component osteosynthesis screw sets which possess greater stability of the osteosynthesis while almost maintaining the dimensions of conventional screws, without enlarging the implant cross-section in the osteotomy/fracture region, were confirmed. To our knowledge, this subject of rotational stability was thus far investigated in only three publications: According to in vitro tests, the recommended placement is as central as possible, orthogonally to the fracture line [16], using the longest possible screw [15], in order to obtain an osteosynthesis which is as stable as possible. As Slade performed in vivo [8] additional augmentation of the in vitro osteosynthesis by a k-wire applied from the distal scaphoid pole to the capitate had no significant effect on the stability of the single screw osteosynthesis in Dodd´[15]. In this publication the scaphoid was not extracted from the wrist, torque was applied by tendon loading and was measured between the scaphoid fragments while instability was displayed kinematically in angular degrees. A further publication proves the dependency of rotational stability on the utilized screw type and attests that the Acutrak^®^ Standard screw has greater rotational stability compared to the Herbert^®^ and an AO 4.0 Cancellous Screw in polyurethane foam (Sawbone^®^) [18]. In this study torque was not applied cyclically, testing material was PU foam with a friction coefficient probably differing from cortico-cancellous bone (from 0.58±0.06 to 0.61±0.07 [23]) and an unphysiological size of a contact surface area was used. That study reports about torque resistance of several screws in relation to each other, no considerations can be drawn on absolute torque values of ex or in vivo specimens.

In view of the clinical relevance, we did not wish to limit ourselves solely to the maximum number of cycles to the specified termination criterion of a 10° rotational clearance in order to systematically evaluate the screw-specific early failures as well. This assessment in the lower torque range up to 200 mNm (Fig 6) is particularly necessary since no in vivo data on actual loads are available, and precise measurements in vivo do not appear technically solvable thus far. Recording implant-specific, torque-dependent clearance of the fragments in rotational twist-out direction with consideration of the number of early failures particularly reflects the screw-specific quality and reliability of the osteosynthesis.

In fact, it was possible to stress a large share of the osteosyntheses in the range of up to 100mNm torque with a low failure rate. However, loads of up to 200 mNm then resulted in clearly visible differences in the stabilization capacity of the various screw types, wherein the BSS long. only showed first signs of weakness at 200 mNm, and the BSS obl. did not show noticeable signs of loosening across this entire load range (Fig 6).

### Technical attributes of the different osteosyntheses

Since the screw base with regard to the screw tip consists of a freely rotating part in the Twinfix^®^ screw, we assume rotational stability nearly solely based on friction adhesion due to bone fragment compression. Due to its construction which is uncoupled from the rotating shaft, the screw base, which rotates freely apart from a small amount of residual friction, prevents rotational instability in the twist-out direction by enlargement of the osteotomy gap, therefore the scaphoid bone fragments consequently remain in contact and continue to maintain friction between the fragments. Distancing of the fragments which is linked to displacement in the twist-out direction and therefore loosening of the screw-fragment connection is expected in all screw models wherein the screw body is rigid within itself. It is likely that this particularly affects the stabilization properties of the HCS screw, which, due to its uniform thread gradient, transforms twist-out movements into enlargements of the osteotomy gap. The initial compression force with which the Twinfix^®^ screw and the HCS^®^ screw compress the fragments depends on the discretion of the surgeon. In contrast to screws with a single, rigid screw body and variable pitches, the compression force is almost solely determined by the construction attributes of the screw itself as well as the bone density. The surgeon may influence the compression force only by the grade of initial fragment reduction and deeper insertion. Since a frictional connection is given only in the screw tip and head areas in central threadless shaft screws, screws with continuous thread were conceived, with the objective of a better development and consistency of the compression and pull out force within the bone [11]. The consistently greater rotational stability of the Acutrak^®^ 2 mini in the lower torque range is maybe due to compacting of cancellous bone between the flanks of the screw threads during the screw insertion process. The progressively changing gradient between the continuous threads causes the screw to clamp and dowel itself within the porous cancellous bone. If this clamping effect decreases due to rotation in the twist-out direction, the osteosynthesis promptly loses rotational stability. Since the extent of compression force [24] is subject to a variation along the fully threaded screw type (Acutrak^®^2 screw), this specific stability attribute may play an important role in clinical application when stabilizing small fragments and scaphoid non unions by means of interpositional bonegrafting [25]. Certain properties which apply to all compression screw osteosyntheses, such as the time-dependent reduction of compression force and thereby the loss of interfragmentary friction adhesion *in vitro* human bone [11,19], as well as the inability of all conventional screws not to prevent rotational movement when friction adhesion is exceeded, led us to develop compression screws with additional construction elements to stabilize the scaphoid synthesis against torsion load.

### New BSS

Both BSS were developed in order to block both fragments against rotation by a second screw which protrudes from the cross-section of the compression screw without significantly enlarging the dimensions of the standard headless screws used in the test.

In spite of the technically immature and clinically not proven stage of prototypes both BSS achieved better measurements in comparison to all standard screws with respect to the secondary outcome but did not resist significantly more stress cycles until failure than the most stable standard screw (Twinfix^®^). In the BSS long., the diameter of the secondary screw and its protrusion from the primary screw needed be increased, as stability against cyclical loading turned out not to be better than the most stable standard screw (primary outcome). BSS obl. clearly demonstrated improved stability of the osteosynthesis along the test sequence. Plastic deformation of both screw elements of the BSS obl. occurred, in the primary screw at the bore for penetration of the secondary screw, the secondary screw due to small shaft diameter. The stability of the BSS long. was superior to the BSS obl. We could not observe plastic deformation of the BSS long. after any test sequences.

## Limitations of This Study

As the study is limited to in vitro manipulation measurements we cannot fully expect compatibility of the set-up to the in vivo situation. As no systemic reaction and healing processes can be mimicked, we can only to some extent rely on simulating the acute osteosynthesis condition and early biomechanical conditions. The scaphoid specimens are limited to aged bones, as cadaver bodies for anatomical research are usually not available from younger donors.

Apart from the rotational direction of scaphoid fragments in scaphoid non unions [26] no in vivo data on the extent of interfragment rotation and on acting forces in vivo situations are described in literature and those will be very difficult to obtain in relevant quality. Therefore we rely on comparability to an already published in vitro study that described investigations in a rotation range of 10° [15]. Concerning acting torque along wrist movements we were forced to rely on published modeling data [9] for a rough estimate.

There is no direct proof from clinical studies or even animal studies if and to what extent interfragmentary rotation influences bone healing or failure of bone healing [5,27]. There are some indirect implications that healing of the scaphoid from a study that concludes that central screw placement has a major positive influence on stability of osteosynthesis of an osteotomized scaphoid [16] and speeds up healing in vivo [17]. Indirect support is given by literature on plate osteosynthesis of long bones that clearly demonstrated a stability dependent consolidation quality of the fractured bone [7]. To our best knowledge there is no available literature on stability depending on the type of screw osteosynthesis in relation to fracture healing.

The actually early development stage of the novel screw models cannot be tested in vivo due to clear ethical limitations. Despite all limitations, a statistically elaborated in vitro study on cadaver specimens can provide more realistic data than one on polyurethane foam (Sawbone^®^).

We selected a planar osteotomy orthogonal to the scaphoid longitudinal axis which was already described in earlier publications as a tested model as our fracture model [16, 22]. Particularly for our inquiry, this can only be compared to clinically found fractures to a limited extent, since it does not offer interlocking fragments, which can result in a certain blocking effect against rotation even in simple compression screw treatment. By using a linear osteotomy in cadaver scaphoids on the other hand, we did not evaluate the effect of comminution, oblique fractures, not completely reduced fractures, cystic type fractures [28], interpositional bonegrafting [24] or cartilage interposition which may decrease the general stability of the fracture in the in vivo situation.

When planning our test protocol, we were aware that even only slight inclinations of the osteotomy plane from orthogonality to the screw axis strongly influence the results, therefore considerable technical efforts were performed to ensure this orthogonality when implementing the scaphoid bone separation and placing the screws.

In our test set up we did not take shear forces perpendicular to the scaphoid longitudinal axis into consideration as it was done in Varga´s finite element study [29]. Torque results also could have been dependent on the depth of potting of the proximal and distal poles of the scaphoid into the negative forms of the scaphoid and sample holder. Excessive torque application by the multiphase motor beyond the intended torque, up to the value of the following increment, was observed. Different variation in bone density and using one single length for different sizes of scaphoids may have influenced the results on stability of the osteosyntheses. In order to guarantee that our measuring results on the evaluation of cycle load bearing capacity were as independent of sample variability as possible, we selected the balanced incomplete random block design and a use of cyclical loading conditions model [22] for the study protocol.

Despite all limitations, we see clear indicators from the study that dual component screw solutions which ensure both compression in the fracture gap and compression-independent stabilization of rotational movements can be used to achieve definite improvements in the stability of scaphoid bone fractures and scaphoid bone non unions.

## Supporting Information

S1 Ethics Commission Statement This file is the decision of ethics commission statement. (PDF)

S1 Table Individual bone mineral density of the scaphoid specimens. (XLS)

S2 Table Specimen and implant individualized cycles during incremental load and torque dependent clearance. (XLS)
