# Supplementary material for: Rotational Stability of Scaphoid Osteosyntheses: An In Vitro Comparison of Small Fragment Cannulated Screws to Novel Bone Screw Sets
Source: PLoS One. 2016 Jun 3;11(6):e0156080. doi: 10.1371/journal.pone.0156080 (PMC4892476; doi:10.1371/journal.pone.0156080)
Supplement: S1 Ethics Commission Statement — (PDF) [file pone.0156080.s001.pdf]

ETHIK-KOMMISSION
DER MEDIZINISCHEN UNIVERSITÄT WIEN
UND DES
ALLGEMEINEN KRANKENHAUSES DER STADT WIEN AKH

Borschkegasse 8b/6 - A-1090 Wien, Austria
☎ 0043 1 404 00 – 2147, 2244 & 🖹 0043 1 404 00 – 1690
E-Mail: ethik-kom@meduniwien.ac.at
www.meduniwien.ac.at/ethik

Sitzung der Ethik-Kommission am 13. Jänner 2009, TOP 128 :

EK Nr: 785/2008
Antragsteller: Dr. Jochen Erhart
Einreichende Institution: Univ.Klin.f. Unfallchirurgie
Projekttitel: Bewegungsinduzierte Kahnbeinbeanspruchung nach Osteosynthese

Die Stellungnahme der Ethik-Kommission erfolgt aufgrund folgender eingereichter Unterlagen:

**Dokument**	**Version/Nr**	**Datiert**
Originalprotokoll:	1	2008-11-20
Kurzfassung:		2008-11-27

Die Kommission fasst folgenden Beschluss (mit X markiert):

☒ Es besteht kein Einwand gegen die Durchführung der Studie.

☐ Die unten bezeichneten Punkte des Antrages sind entweder noch unerledigt bzw sollten von den Antragstellern geändert/ nachgereicht werden. Nach entsprechender Vorlage/Erledigung kann auch vor der nächsten Ethik-Kommissions Sitzung ein endgültig positiver Beschluss ausgefertigt werden. Der Antrag wird in der nächsten Sitzung der Kommission nicht mehr behandelt.
Achtung: Werden die geforderten Unterlagen von den Antragstellern nicht innerhalb von 3 Sitzungsperioden (ab Datum dieser Sitzung) nachgereicht, gilt der Antrag ohne weitere Benachrichtigung als zurückgezogen und muss gegebenenfalls als Neuantrag eingereicht werden.

☐ Es bestehen Einwände gegen die Durchführung der Studie in der eingereichten Form. Die unten angeführten Punkte sollten von den Antragstellern entsprechend geändert und der Kommission neu vorgelegt werden. Der Antrag wird in der nächsten Sitzung der Kommission nochmals behandelt.
Achtung: Werden die geforderten Unterlagen von den Antragstellern nicht innerhalb von 3 Sitzungsperioden (ab Datum dieser Sitzung) nachgereicht, gilt der Antrag ohne weitere Benachrichtigung als zurückgezogen und muß gegebenenfalls als Neuantrag eingereicht werden.

☐ Der Antrag wird von der Ethik-Kommission abgelehnt.

☐ Der TOP wird bis zur nächsten Sitzung vertagt (Begründung siehe unten)

Kommentare:

Zum Prüfplan :

Zur Patienteninformation :

Zur Versicherungsbestätigung : **nicht erforderlich**

Andere : **Weder aus dem Antragsformular noch aus dem Protokoll geht hervor, wo die Messungen stattfinden bzw woher die Leichen stammen. Sollten die Leichen frisch Verstorbener auf der Pathologie verwendet werden, so müsste auch ein Pathologe der Medizinischen Universität Wien involviert werden.**
Weiters sollte noch in Punkt 7.6 des Antragsformulars ein Satz hinzugefügt werden, dass es zu keiner Verunstaltung der Leichen kommt, falls es sich nicht um Leichen der Anatomie handelt.

<u>Nachtrag vom 30. Jänner 2009</u>: Die Antragsteller legen am 29.1.09 eine Stellungnahme vor, aus der ersichtlich ist, dass bei vorliegendem Antrag Leichenunterarme der Abteilung f. angewandte Anatomie betroffen sind.

Die Ethik-Kommission geht - rechtlich unverbindlich – davon aus, daß es sich um keine klinische Prüfung gemäß AMG/MPG handelt.

Mitgliederliste der Ethik-Kommission (aktueller Stand am Sitzungstag) beiliegend. Mitglieder der Ethik-Kommission, die für diesen Tagesordnungspunkt als befangen anzusehen waren und daher laut Geschäftsordnung an der Entscheidungsfindung/Abstimmung nicht teilgenommen haben: **keine**

Univ.Prof.Dr. Ernst Singer
Vorsitzender der Kommission

ACHTUNG: Unter Berücksichtigung der „ICH-Guideline for Good Clinical Practice“ gilt dieser Beschluß **<u>ein Jahr ab Datum der Ausstellung.</u>** Gegebenenfalls hat der Antragsteller eine Verlängerung der Gültigkeit mittels Formular für „Meldungen“ rechtzeitig vorzulegen.
